# Sleep Indices and Cardiac Autonomic Activity Responses during an International Tournament in a Youth National Soccer Team

**DOI:** 10.3390/ijerph18042076

**Published:** 2021-02-20

**Authors:** Pedro Figueiredo, Júlio Costa, Michele Lastella, João Morais, João Brito

**Affiliations:** 1 Portugal Football School, Portuguese Football Federation, FPF, 1495-433 Cruz Quebrada, Portugal; julio.costa@fpf.pt (J.C.); joao.m.morais@fpf.pt (J.M.); joao.brito@fpf.pt (J.B.); 2 Research Center in Sports Sciences, Health Sciences and Human Development, CIDESD, University Institute of Maia, ISMAI, 4475-690 Maia, Portugal; 3 Center of Research, Education, Innovation and Intervention in Sport, CIFI2D, Faculty of Sport, University of Porto, 4200-450 Porto, Portugal; 4 Appleton Institute for Behavioural Science, Central Queensland University, Adelaide, QLD 5034, Australia; m.lastella@cqu.edu.au

**Keywords:** accelerometer, adolescent, football, match, parasympathetic system, recovery, sleep duration, sleep quality, training load

## Abstract

This study aimed to describe habitual sleep and nocturnal cardiac autonomic activity (CAA), and their relationship with training/match load in male youth soccer players during an international tournament. Eighteen elite male youth soccer players (aged 14.8 ± 0.3 years; mean ± SD) participated in the study. Sleep indices were measured using wrist actigraphy, and heart rate (HR) monitors were used to measure CAA during night-sleep throughout 5 consecutive days. Training and match loads were characterized using the session-rating of perceived exertion (s-RPE). During the five nights 8 to 17 players slept less than <8 h and only one to two players had a sleep efficiency <75%. Players’ sleep duration coefficient of variation (CV) ranged between 4 and 17%. Nocturnal heart rate variability (HRV) indices for the time-domain analyses ranged from 3.8 (95% confidence interval, 3.6; 4.0) to 4.1 ln[ms] (3.9; 4.3) and for the frequency-domain analyses ranged from 5.9 (5.6; 6.5) to 6.6 (6.3; 7.4). Time-domain HRV CV ranged from 3 to 10% and frequency-domain HRV ranged from 2 to 12%. A moderate within-subjects correlation was found between s-RPE and sleep duration [r = −0.41 (−0.62; −0.14); *p* = 0.003]. The present findings suggest that youth soccer players slept less than the recommended during the international tournament, and sleep duration was negatively associated with training/match load.

## 1. Introduction

Sleep is considered an essential component for athlete recovery due to its physiological and psychological restorative effects [1]. Sleep habits are commonly characterized by sleep duration and quality [2]. The National Sleep Foundation indicates that a sleep duration <8 h and sleep efficiency—defined as the percentage of time in bed that is spent asleep—below 75% per night are considered inappropriate for teenagers (aged 14–17 years) to maintain adequate levels of performance, learning, development and physical and mental health [2,3]. 

On athletes, studies have mostly focused on the negative effect of inadequate sleep duration on athletic performance and recovery [4,5]. Although some studies have described sleep efficiency a key indicator of sleep quality [6,7] in elite athletes [8], its impact on health and performance indicators have been less explored. Additionally, the relationship of sleep habits with exercise and athletic training is complex and reciprocal [9]. Therefore, the associations between training and matches loads with both sleep quantity and quality need to be explored.

Recently, studies exploring the potential impact of training load on sleep habits have included measures of heart rate (HR) variability (HRV) during sleep, which reflects cardiac parasympathetic modulation [10]. HRV is sensitive to fatigue due to increased training loads [11] and has been also useful in evaluating individual training-induced stress and perturbation of allostasis [12,13]. Considering that parasympathetic activity is high during night sleep, night recordings may allow better discrimination of the changes in autonomic nervous systems equilibrium [14,15]. Additionally, HR may increase during the spontaneous electroencephalographic arousals throughout the night and by short- or long-term awakenings [16]. 

Periods of intensified training loads have been shown to increase the level of disturbance in sleep [17] and HRV [18], and it is well-known that changes in nocturnal HRV after exercise may last up to 24 h [19]. However, studies describing the variation on nocturnal HRV after exercise (i.e., training and/or competition) sessions, especially in young athletes, are lacking. Additionally, studies exploring the associations between training and matches loads with nocturnal HRV are scarce, and none have studied young soccer players [20]. 

Finally, most previous studies have focused on group data to analyze sleep and HRV changes in soccer players [21,22,23,24,25], which has limited capacity to detect individual responses. Although individual analysis methods have recently been used to track individual changes [20,26,27], these methods have not been used to analyze data on sleep and nocturnal HRV variability in youth soccer players. 

In the current study, we aimed to: (1) describe the individual patterns of sleep and nocturnal cardiac autonomic activity, and (2) to explore the intra-individual associations of training/match load with sleep and HRV measures in male youth soccer players during an international tournament. We hypothesized that athletes would present a high inter- and intra-variability in sleep and HRV measures. Additionally, we expected a negative within-subject association of sleep and HRV with training/match load.

## 2. Materials and Methods 

### 2.1. Participants 

Eighteen elite youth male soccer players (aged 14.8 ± 0.3 years; height: 1.76 ± 0.07 cm; body mass: 66.1 ± 6.3 kg; mean ± SD) from the Portuguese U-16 National team volunteered to participate in this study. The study design was carefully explained to the subjects, and written informed consent was obtained from parents and/or legal guardians. The study followed the Declaration of Helsinki and was approved by the Ethics Committee of the Faculty of Sports, University of Porto (CEFADE 44.2019).

### 2.2. Procedures 

**Figure 1:**
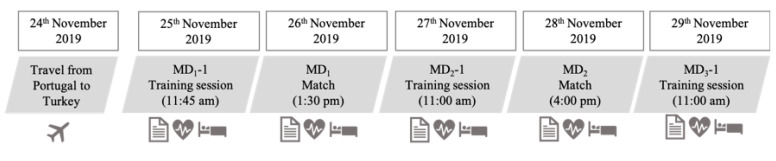
Schematic showing the study design. Session-rating of perceived exertion included training and match days. During night-sleep of the 5 days, cardiac autonomic activity and sleep characteristics were assessed using heart rate monitors and accelerometers, respectively.

Data collection was performed throughout 5 consecutive days (3 training sessions and 2 matches) during an international tournament held in Turkey (Figure 1). Players habitual sleep and nocturnal cardiac autonomic activity were monitored every night. The players stayed in the same hotel and slept in twin rooms allocated by the technical staff. All training sessions were conducted outdoor in a natural grass pitch. The two competitive matches were held in the same stadium located at the Emirhan Sport Center (Antalya, Turkey). All facilities were closely located, and thus travel fatigue was avoided. Training schedules were set by the team coaching staff, with no interference by the research team (or with other routines, including habitual sleep habits). The players followed their normal dietary routine during the data collection period. 

During each night-sleep of the tournament, players wore a 3-axial accelerometer (Actigraph LLC wGT3X-BT, Pensacola, FL, USA) on the non-dominant wrist during each night-sleep. Data were analyzed using corporate software (ActiLife LLC Pro software v6.13.3, Pensacola, FL, USA). The sampling frequency was 50 Hz and the epoch of activity counts was 60 s [28]. Accelerometer data were extracted using the Sadeh’s (S) algorithm—originally validated on a healthy sample of adolescents and young adults (age range 10–25 years) [28]. Sleep outcomes included sleep duration (amount of sleep hours) and sleep efficiency (percentage of time in bed that was spent asleep) [20,28] were analyzed according to the National Sleep Foundation guidelines [2]. A sleep duration <8 h was considered an indicator of inappropriate sleep quantity, and a sleep efficiency ≤74% was considered an inappropriate sleep quality.

Players wore HR monitors (Firstbeat Bodyguard2^®^, Firstbeat Technologies, Helsinki, Finland) to record the cardiac autonomic activity during night-sleep. Data were analyzed using the SWS episode method, which accounts for the deep stage of sleep [12]. This method records 10 min of normal R-R intervals [29]. The natural logarithm of the square root of the mean of the sum of the squares of differences between adjacent normal R-R intervals (lnRMSSD; vagal modulation index) was used as the main HRV outcome of the time domain analyses to understand nocturnal changes in cardiac autonomic activity [12,29]. Fast Fourier Transform (Welch’s periodogram: 300-s window with 50% overlap) [30] was used to obtain measures of nocturnal cardiac autonomic activity in the frequency-domain, considering both low frequency (LF: 0.004–0.15 Hz) and high frequency (HF: 0.15-0.4 Hz) indices [30]. For frequency analyses, R-R trend components were removed using an advanced smoothness prior approach, with a smoothing parameter of λ = 500, which corresponds to a cut-off frequency of 0.035 Hz [30]. Ratio (i.e., LF/HF) indices were calculated from the non-transformed LF and HF data [31]. R-R recordings were exported using the Kubios version 3.2 Heart Rate Variability software (Biosignal Analysis and Medical Imaging Group at the Department of Applied Physics, University of Kuopio, Kuopio, Finland).

Training and match loads were quantified by session-rating of perceived exertion (s-RPE) to characterize training practices and competition demands during the observation period. Training load data were collected and recorded by a member of the team’s medical staff. Players reported individual RPE using the Borg category ratio scale (CR10) after each training session or match. All athletes were familiar with the CR10 method. The session or match load was determined by the individual CR10 score multiplied by the individual exposure time (training and match volume) [32].

### 2.3. Statistical Methods 

Sample distribution was tested using the Shapiro–Wilk test for sleep quality and efficiency, HRV, and training and match load for each day of the tournament. Variables are presented as mean with the 95% confidence interval (CI) unless otherwise stated. The coefficient of variation (CV; CV = [standard deviation/mean] × 100) was calculated for the whole group and individually for sleep duration and HRV indices (i.e., lnRMSSD, lnLF, LnHF and LF/HF) across the 5 days to analyze variability. 

Linear mixed model and generalized linear mixed model analysis were performed to examine differences in sleep duration, sleep efficiency, and nocturnal HRV indices across the 5 days of data collection. An α-level of 0.05 was set as the level of significance for statistical comparisons. The days with training sessions and matches were included as a fixed effect and player identity (subject ID) as the random effect. The variance-covariance structures were selected according to the smallest Akaike Information Criterion. Bonferroni pairwise comparisons were used to test the day-to-day mean differences for sleep duration, sleep efficiency, and nocturnal HRV indices. 

We tested the within-subjects correlations (r, 95% CI) [33] between: sleep duration and efficiency, both sleep indices and HRV indices, both sleep indices and s-RPE, and finally HRV indices and s-RPE. The correlations with sleep duration were adjusted (partial within-subjects correlations) for sleep efficiency and vice-versa [34]. We qualitatively interpreted the magnitudes of correlation using the following criteria: *trivial* (r ≤ 0.1), *small* (r = 0.1–0.3), *moderate* (r = 0.3–0.5), *large* (r = 0.5–0.7), *very large* (r = 0.7–0.9) and *almost perfect* (r ≥ 0.9) [35]. When the 95% CI overlapped positive and negative values, the effect was deemed to be *unclear*.

All statistical analyses were conducted using the lme4, lsmeans and rmcorr packages in R statistical software (version 3.4.1, R Foundation for Statistical Computing, Vienna, Austria). 

## 3. Results 

The athletes’ habitual sleep characteristics, nocturnal cardiac autonomic activity and the training/matches load per day of data collection are summarized in Table 1. Match days occurred on days 2 and 4. On average, sleep duration ranged between 6.7 (6.3; 7.1) to 7.5 h (7.0; 7.7), and sleep efficiency ranged between 82 to 84%. The first match day (MD_1_) had the lowest average sleep duration (6.7 h ), which was only significantly different from the sleep duration in MD_1_-1 (7.5 h, *p* = 0.03). No differences in sleep efficiency were found between the 5 days. The highest training/match load was observed in MD_2_ [469 AU (319; 689)], while the lowest load was recorded in day 1 and 3. Players had a constant average HR throughout the 5-day period (51 ± 6 bpm; mean ± SD), and no differences were observed in all HRV indices. Due to technical problems and/or player compliance, we had the following missing data: actigraphy *n* = 5 (6%), HRV *n* = 3 (4%), s-RPE *n* = 6 (8%).

**Figure 2:**
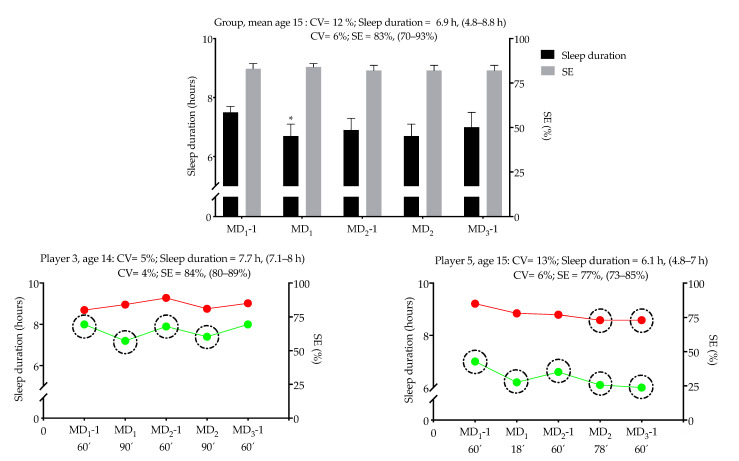
Analytical graph of the group (mean and 95% confidence interval) and individual sleep duration and sleep efficiency (SE) values of two players during the 5 days of the tournament. Averages, maximum and minimum values are also presented. Green and red dots represent daily changes in sleep duration and SE, respectively. The black dashed circumferences represent the days where sleep duration and SE were lower than the recommended amounts (i.e., sleep duration < 8 h and SE < 75%). Abbreviations: MD, Match-day; TST, total sleep time; CV, coefficient of variation. * Significantly different from MD_1_-1 (*p* = 0.03).

Figure 2 displays the group (*n* = 18) and individual sleep data of two players. The individual sleep data for all players is presented in Figure S1 (Supplementary Materials). The recommended cut-point of 8 h/night in the 5 nights was not reached by several players: MD_1_-1 (*n* = 16), MD_1_ (*n* = 17), MD_2_-1 (*n* = 13), MD_2_ (*n* = 14), and MD_3_-1 (*n* = 8). In MD_1_-1 and MD_1_ only two players had a sleep efficiency ≤74%, and in MD_2_ and MD_3_-1 only one player in each night did not reach the sleep efficiency threshold. Sleep duration CV ranged between 4 to 17%, while sleep efficiency ranged between 1 to 10% across the 5 days (Figure S1). Differences in sleep duration and SE patterns between athletes can be observed in the two figures displaying the data for players 3 and 5. 

**Figure 3:**
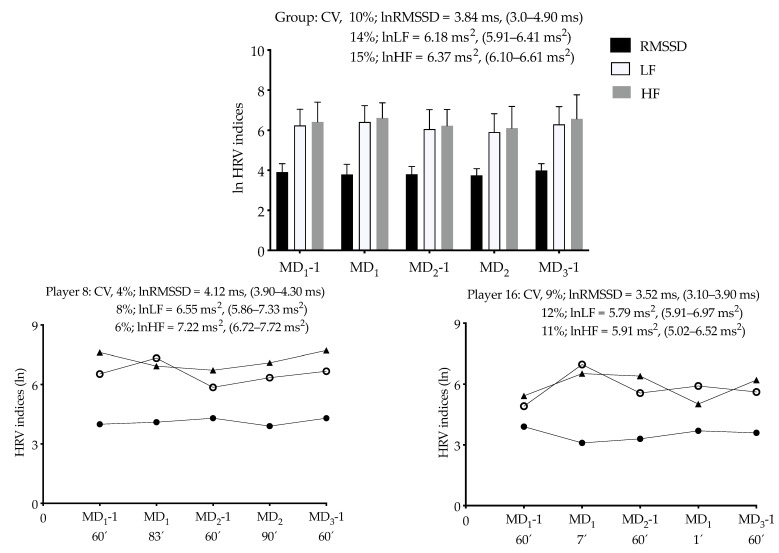
Analytical graph of the group (mean and 95% confidence interval) and individual cardiac parasympathetic activity (measured as natural logarithm of the root mean square of successive R-R intervals (lnRMSSD), natural logarithm of low frequency (lnLF), and as natural logarithm of high frequency (lnHF)) values of two players for each day of the tournament. Averages, maximum and minimum values are also presented. Abbreviations: MD, Match-day; CV, coefficient of variation.

Figure 3 shows the group (*n* = 18) and individual nocturnal cardiac autonomic activity data of two players. The individual data (assessed by overnight lnRMSSD, lnLF and lnHF) for all players is presented in Figure S2 (Supplementary Materials). As a group, the cardiac autonomic activity was stable during the 5-day period, while the individual lnRMSSD, lnLF, lnHF and LF/HF CVs ranged between 3 and 10%, 2 and 12%, 2 and 12%, 11 and 16%, respectively (Figure S3). Differences in HRV indices fluctuations can be observed in the two figures displaying the data of players 8 and 16.

### 3.1. Within-Subject Correlation between Sleep Indices and Nocturnal HRV

The unadjusted within-subject correlation analysis revealed an *unclear* correlation between nocturnal HRV indices and both sleep duration and efficiency (Table 2). However, after adjusting for sleep efficiency, a *moderate* positive correlation between sleep duration and lnRMSSD [*r* = 0.34 (0.11; 0.53); *p* = 0.008] and a *small* positive correlation between sleep duration and lnHF [*r* = 0.28 (0.05; 0.48); *p* = 0.03] were found. The remaining correlations were similar to the unadjusted findings. Adjusting for sleep duration, a *small* negative association between sleep efficiency and lnRMSSD [*r* = −0.25 (−0.46; −0.02); *p* = 0.04] was observed. The remaining correlations were similar to the unadjusted findings.

A *large* positive within-subject correlation was found between both sleep parameters [*r* = 0.59 (0.38; 0.74); *p* < 0.001].

### 3.2. Within-Subject Correlation between Sleep Indices and Nocturnal HRV with Training/Match Load

The within-subject correlations of the night-sleep variables and nocturnal HRV indices with s-RPE during the 5 days of tournament are presented in Table 3. A *moderate* negative correlation was found only between s-RPE and sleep duration [r = −0.41 (−0.62; −0.14); *p* = 0.003]. This result remained almost unchanged after controlling for the sleep efficiency [r = −0.36 (−0.55; −0.13); *p* = 0.006]. The adjusted correlations between the remaining variables were similar to the unadjusted results.

## 4. Discussion 

This observational study describes habitual sleep and nocturnal cardiac autonomic activity, and their relationship with training/match load in male youth soccer players during an international tournament, using non-invasive and time-efficient methods. We found that most players slept less than the recommended 8 h/night during the tournament, independently of being training session-day or match-day. Another key finding was the negative association between training/match load (s-RPE) and sleep duration. Our findings support the need to develop and implement individual sleep strategies, due the known negative consequences of reduced sleep time or sleep deprivation on young athletes’ health and performance [36]. Finally, the training and matches demands did not affect players’ nocturnal cardiac autonomic activity, demonstrated by the small fluctuations in HRV indices. Therefore, we confirmed our first hypothesis as our data showed that youth soccer players presented a high variability in sleep and HRV measures during an international tournament. However, our second hypothesis was only partially confirmed, as only sleep duration was negatively associated with training/match load.

During the 5-day tournament, sleep duration was relatively constant at the group level, except for the first match day (night 2) that registered the lowest amount of sleep (particularly compared with the previous night). Of note, the average sleep duration was below the recommend cut-point (8 h/night) from the National Sleep Foundation [3] and more recently by the Sleep Consensus Recommendations for athletes [37]. Youth athletes value their social time with friends, and the use of electronic devices, which may impact their sleep [38,39]. Thus, it is suggested that strategies to extend sleep duration to achieve the recommended levels can improve performance, mood, and stress levels [37]. 

Overall, players obtained more sleep on the nights before the match-day, which had the lowest s-RPE. This is consistent with previous studies that reported that sleep quantity may be affected by the type of training day within the schedule [25,40] and that elite players obtain a reduced amount of sleep on the night of the match compared with the nights of training days [41]. This link between type of day and sleep duration may be related to the preparation strategies for the match, as players may recognize the benefits of longer sleep time for recovery and performance [42], as well as the influence of the match on match-day. This finding is also corroborated by the observed *moderate* correlation between sleep duration and s-RPE in both, the unadjusted and adjusted models. Periods of intensified training loads have been associated with higher disturbance levels in sleep [17]. This occurrence may be a result of overreaching, increased levels in muscular soreness [17], and pro-inflammatory responses [43]. Similarly, high training loads (such as soccer match) are also likely to induce similar physiological responses [44]. Further research is required to determine the physiological effects of the observed relationship and how it may impact recovery and performance.

Although sleep efficiency seemed less affected by training/match load, we found a *large* positive within-subject correlation between sleep efficiency and sleep duration. Our finding reinforces that strategies targeting sleep duration can have additional benefits in other sleep characteristics. The impact of sleep efficiency in health and athletic performance has been poorly explored.

In the current study, no significant changes in HRV indices across the 5-day tournament and an *unclear* within-subject correlation with s-RPE were observed. Our finding is in line with the results described in elite female national soccer players [20]. Other studies also reported that the fitter young soccer players with greater exercise tolerance had lower daily allostatic perturbation, leading to smaller daily changes in lnRMSSD, and in turn, lower CV values, as well as reduced perceived fatigue [45]. A higher resilience to sustained elevated training and match loads without presenting signs of severe nocturnal cardiac autonomic perturbation, and a higher readiness to perform [46] may, in part, explain these results. More research is needed in this topic.

Finally, we also observed a *small* and *moderate* positive correlation (adjusted for sleep efficiency) observed between HRV indices (i.e., lnHF and lnRMSSD, respectively) and sleep duration. Few studies have explored this association, but findings are inconsistent [18,47]. Thus, the link between sleep and overnight HRV indices (i.e., time and frequency domain analyses) remains unclear.

The strengths of our study are the longitudinal study design, individual and within-subjects analysis, and the use of wrist-worn accelerometers and HR monitors, which have been validated against polysomnography [48] and standard electrocardiogram equipment to detect heartbeats [49], respectively. However, findings should be interpreted in light of the study limitations. Some potential factors that could have influenced both sleep duration and quality were not controlled in the present study, such as changes in hormonal levels, sleep disorders, nap time, use of caffeine, level of light exposure during daytime, differences in room temperature, use of electronic devices, traveling, and sleeping in a hotel room. However, this observational study was set in a real-world scenario, which limits the access to some of those measurements. Finally, other potential limitations are the possible influence of missing data on the presented CV values, the lack of a time-point with no training or matches (baseline) and autonomic tests.

## 5. Conclusions

The results of this study indicate that young soccer players’ sleep duration and efficacy may be affected by training and match demands during a tournament. Overall, this study highlights the high variability of sleep indices, that youth soccer players slept less than the recommended levels, and sleep duration was negatively associated with workloads. Finally, it remains to be determined whether such declines in sleep duration are detrimental to recovery, and the magnitude of benefits that can be achieved when the individual sleep needs are met.

## Figures and Tables

**Table 1:** Players’ actigraphy sleep characteristics, nocturnal cardiac autonomic activity, and session-rating of perceived exertion (s-RPE) during the 5 consecutive days of tournament.

Variables	MD_1_-1	MD_1_	MD_2_-1	MD_2_	MD_3_-1
Sleep duration (h)	7.5 (7.0; 7.7)	6.7 (6.3; 7.1) *	6.9 (6.5; 7.3)	6.8 (6.3; 7.1)	7.0 (6.6; 7.5)
Sleep efficiency (%)	83 (81; 86)	84 (81; 86)	82 (79; 85)	82 (79; 85)	82 (79; 85)
lnRMSSD (ms)	3.9 (3.7; 4.1)	3.8 (3.6; 4.0)	3.9 (3.7; 4.1)	3.8 (3.6; 4.0)	4.1 (3.9; 4.3)
lnLF (ms^2^)	6.2 (5.8; 6.7)	6.4 (6.1; 7.0)	6.1 (5.7; 6.6)	5.9 (5.6; 6.5)	6.3 (6.0; 7.0)
lnHF (ms^2^)	6.4 (5.9; 6.9)	6.6 (6.2; 7.2)	6.2 (5.9; 6.8)	6.1 (5.8; 6.7)	6.6 (6.3; 7.4)
LF/HF	0.9 (0.9; 1.1)	0.9 (0.9; 1.1)	0.9 (0.9; 1.0)	0.9 (0.9; 1.1)	0.9 (0.9; 1.0)
s-RPE (AU)	233 (170; 320)	388 (275; 548) *	225 (161; 314) ^#^	469 (319; 689) *^†^	251 (168; 375) ^‡^

Values are group mean and 95% confidence interval estimates. * Significantly different from MD_1_-1. **^#^** Significantly different from MD_1_. ^†^ Significantly different from MD_2_-1. ^‡^ Significantly different from MD_2_. Abbreviations: lnRMSSD, natural logarithm of square root of the mean of the sum of the squares of differences between adjacent NN intervals; lnLF, natural logarithm of low frequency; lnHF, natural logarithm of high frequency; LF/HF, ratio of the low to high frequency power; AU, arbitrary units.

**Table 2:** Within-subject correlation between players’ sleep indices and nocturnal cardiac autonomic activity during the 5-days tournament (n = 18).

	Sleep Duration			Sleep Efficiency		
	*r* (95% CI)	*p*	Description	*r* (95% CI)	*p*	Description
lnRMSSD	0.22 (−0.05; 0.47)	0.11	*Unclear*	−0.12 (−0.38; 0.16)	0.38	*Unclear*
lnLF	0.14 (−0.14; 0.40)	0.32	*Unclear*	−0.02 (−0.29; 0.26)	0.91	*Unclear*
lnHF	0.22 (−0.06; 0.46)	0.12	*Unclear*	−0.04 (−0.31; 0.24)	0.78	*Unclear*
LF/HF	−0.002 (−0.38; 0.15)	−0.12	*Unclear*	−0.002 (−0.27; 0.27)	0.98	*Unclear*

lnRMSSD, natural logarithm of square root of the mean of the sum of the squares of differences between adjacent NN intervals; lnLF, natural logarithm of low frequency; lnHF, natural logarithm of high frequency; LF/HF, ratio of the low to high frequency power; CI, confidence Interval.

**Table 3:** Within-subject correlation between sleep indices and nocturnal cardiac autonomic activity with session-rating of perceived exertion (s-RPE) during the 5-day tournament (n = 18).

	s-RPE		
	*r* (95% Confidence Interval)	*p*	Description
Sleep duration	−0.41 (−0.62; −0.14)	0.003	*Moderate*
Sleep efficiency	−0.08 (−0.35; 0.21)	0.59	*Unclear*
lnRMSSD	−0.24 (−0.48; 0.04)	0.08	*Unclear*
lnLF	−0.12 (−0.38; 0.16)	0.34	*Unclear*
lnHF	−0.16 (−0.41; 0.12)	0.27	*Unclear*
LF/HF	−0.03 (−0.30; 0.25)	0.82	*Unclear*

lnRMSSD, natural logarithm of square root of the mean of the sum of the squares of differences between adjacent NN intervals; lnLF, natural logarithm of low frequency; lnHF, natural logarithm of high frequency; LF/HF, ratio of the low to high frequency power.

## Data Availability

Data cannot be shared publicly because it contains potentially sensitive information and have been obtained from a third party (i.e. Portugal Football School, Portuguese Football Federation) and access restrictions apply. Data are available from the Data Protection Office, Portuguese Football Federation (Data Access contact via e-mail: dpo@fpf.pt) for researchers who meet the criteria to access confidential data.

## Supplementary Materials

The following are available online at https://www.mdpi.com/1660-4601/18/4/2076/s1, Figure S1: Individual sleep indices data for all players during the 5 days of the tournament. Figure S2: Individual nocturnal heart rate variability indices data for all players during the 5 days of the tournament.
