# Supplementary material for: Sleep Indices and Cardiac Autonomic Activity Responses during an International Tournament in a Youth National Soccer Team
Source: Int J Environ Res Public Health. 2021 Feb 20;18(4):2076. doi: 10.3390/ijerph18042076 (PMC7924379; doi:10.3390/ijerph18042076)
Supplement: Supplementary file 1 [file ijerph-18-02076-s001.zip › Supplement file 2.pdf]

Supplement file 2.

Player 1: CV, 7%; lnRMSSD = 3.82 ms, (3.50–4.10 ms)
12%; lnLF = 5.43 ms^2, (4.41–6.30 ms^2)
9%; lnHF = 6.46 ms^2, (5.70–7.18 ms^2)

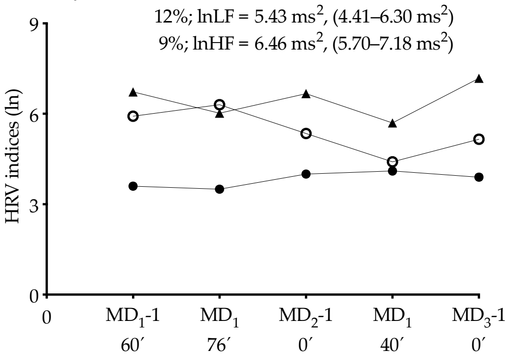

Player 2: CV, 4%; lnRMSSD = 3.98 ms, (3.90–4.20 ms)
5%; lnLF = 6.84 ms^2, (6.42–7.30 ms^2)
4%; lnHF = 6.77 ms^2, (6.52–7.09 ms^2)

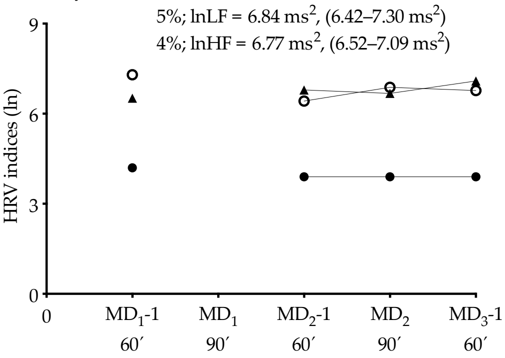

Player 3: CV, 10%; lnRMSSD = 3.74 ms, (3.20–4.20 ms)
12%; lnLF = 5.92 ms^2, (5.47–7.38 ms^2)
12%; lnHF = 6.09 ms^2, (5.08–6.62 ms^2)

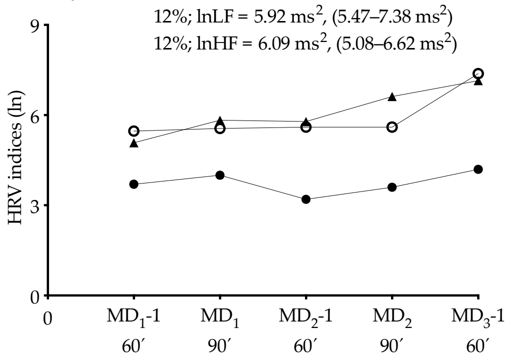

Player 4: CV, 3%; lnRMSSD = 4.80 ms, (4.70–4.90 ms)
9%; lnLF = 7.47 ms^2, (6.99–7.95 ms^2)
4%; lnHF = 8.03 ms^2, (7.79–8.27 ms^2)

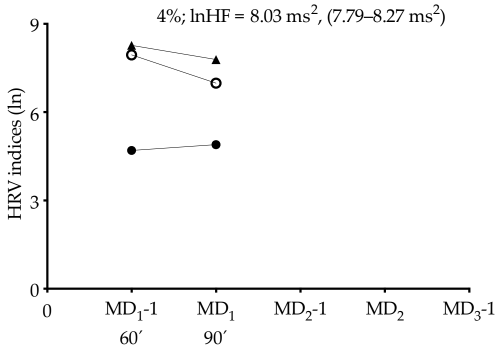

Player 5: CV, 4%; lnRMSSD = 3.40 ms, (3.30–3.60 ms)
5%; lnLF = 4.78 ms^2, (4.47–5.15 ms^2)
8%; lnHF = 4.90 ms^2, (4.66–5.52 ms^2)

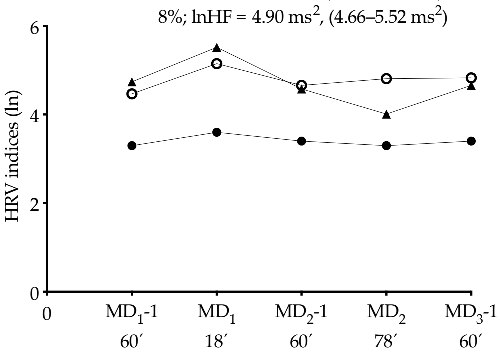

Player 6: CV, 6%; lnRMSSD = 3.57 ms, (3.40–3.80 ms)
9%; lnLF = 5.93 ms^2, (5.27–6.52 ms^2)
9%; lnHF = 5.79 ms^2, (5.43–6.57 ms^2)

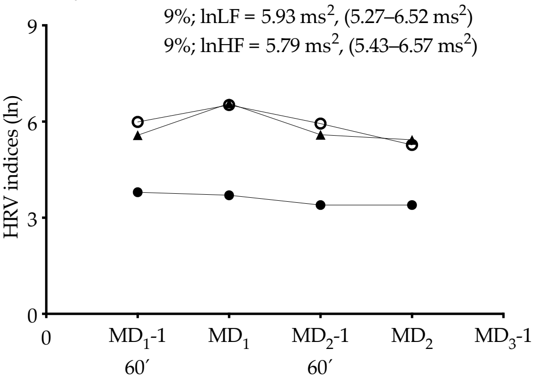

Player 7: CV, 3%; lnRMSSD = 4.03 ms, (3.90–4.20 ms)
5%; lnLF = 7.31 ms^2, (6.84–7.71 ms^2)
2%; lnHF = 6.97 ms^2, (6.86–7.19 ms^2)

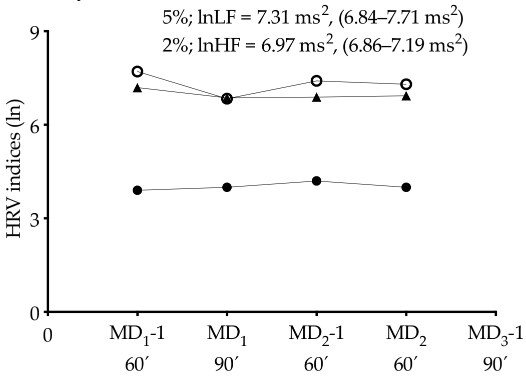

Player 8: CV, 4%; lnRMSSD = 4.12 ms, (3.90–4.30 ms)
8%; lnLF = 6.55 ms^2, (5.86–7.33 ms^2)
6%; lnHF = 7.22 ms^2, (6.72–7.72 ms^2)

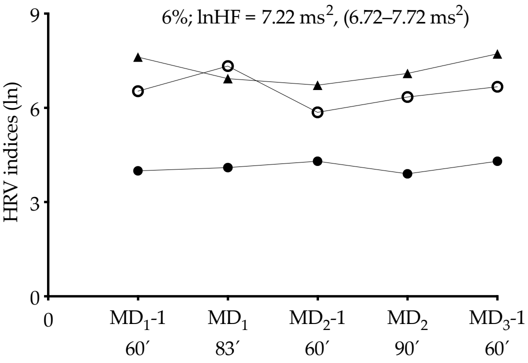

Player 9: CV, 7%; lnRMSSD = 3.67 ms, (3.30–4.0 ms)
8%; lnLF = 7.03 ms^2, (6.32–7.62 ms^2)
8%; lnHF = 7.16 ms^2, (6.58–7.33 ms^2)

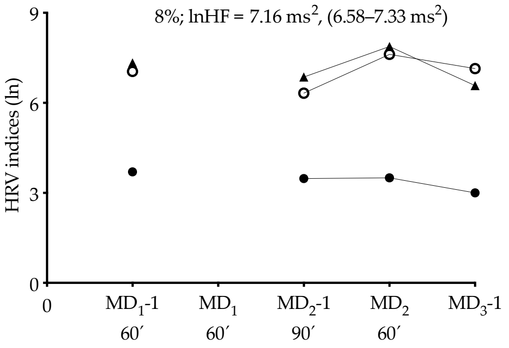

Player 10: CV, 6%; lnRMSSD = 4.40 ms, (4.20–4.60 ms)
11%; lnLF = 7.06 ms^2, (6.53–7.59 ms^2)
11%; lnHF = 7.63 ms^2, (7.02–8.23 ms^2)

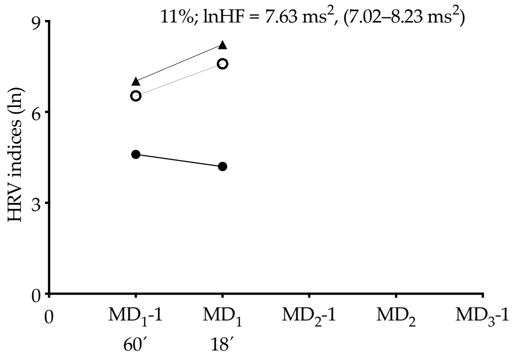

Player 11: CV, 10%; lnRMSSD = 3.94 ms, (3.60–4.60 ms)
12%; lnLF = 5.80 ms^2, (5.28–6.90 ms^2)
12%; lnHF = 6.77 ms^2, (6.19–8.14 ms^2)

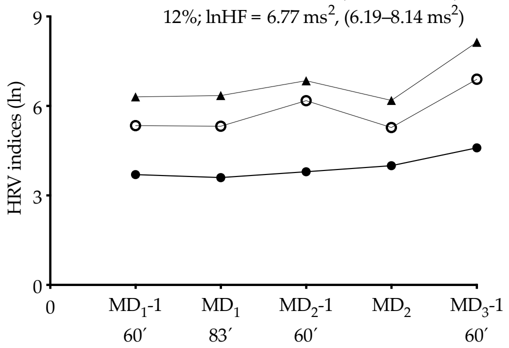

Player 12: CV, 7%; lnRMSSD = 3.60 ms, (3.40–3.80 ms)
2%; lnLF = 6.76 ms^2, (6.85–6.67 ms^2)
9%; lnHF = 6.60 ms^2, (6.17–7.02 ms^2)

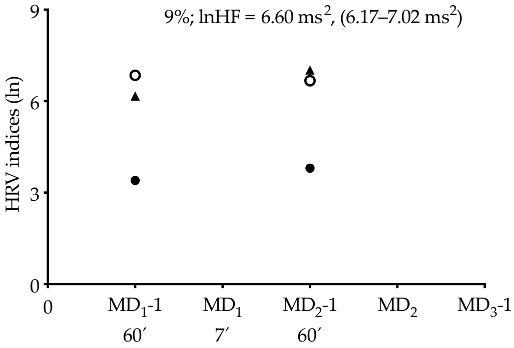

Player 13: CV, 9%; lnRMSSD = 3.42 ms, (3.00–3.80 ms)
12%; lnLF = 5.70 ms^2, (4.97–6.72 ms^2)
12%; lnHF = 4.94 ms^2, (4.26–5.70 ms^2)

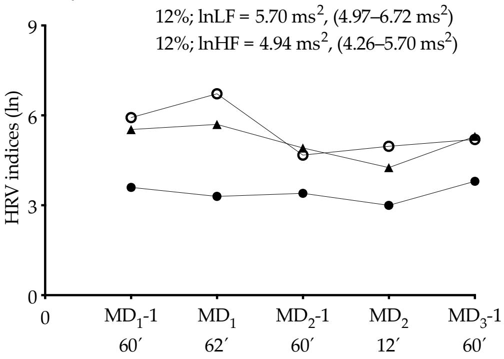

Player 14: CV, 7%; lnRMSSD = 4.25 ms, (3.90–4.60 ms)
5%; lnLF = 7.03 ms^2, (7.03–7.42 ms^2)
9%; lnHF = 7.09 ms^2, (6.40–7.83 ms^2)

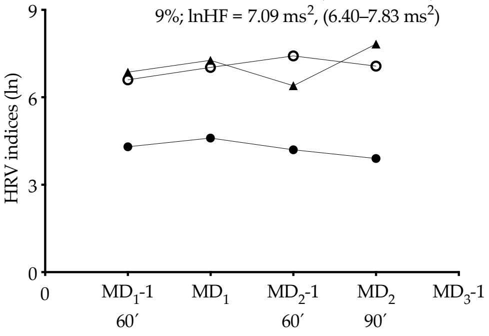

Player 15: CV, 7%; lnRMSSD = 3.78 ms, (3.50–4.10 ms)
7%; lnLF = 6.67 ms^2, (6.08–7.21 ms^2)
7%; lnHF = 6.54 ms^2, (6.0–7.15 ms^2)

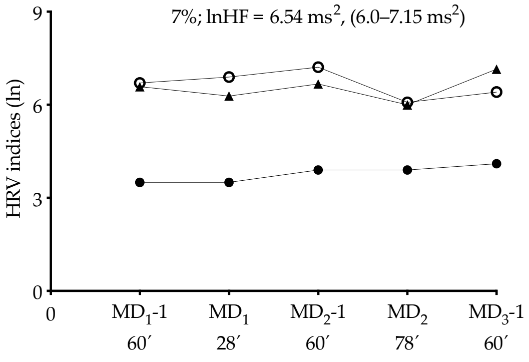

Player 16: CV, 9%; lnRMSSD = 3.52 ms, (3.10–3.90 ms)
12%; lnLF = 5.79 ms^2, (5.91–6.97 ms^2)
11%; lnHF = 5.91 ms^2, (5.02–6.52 ms^2)

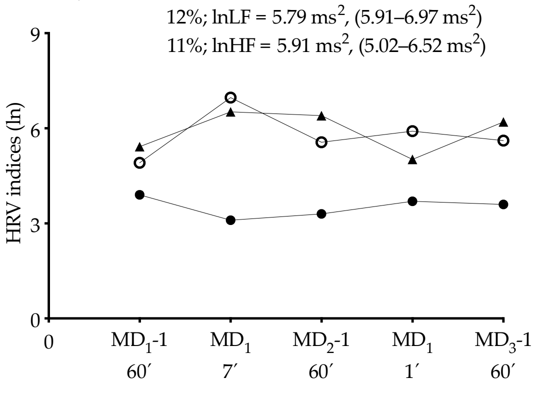

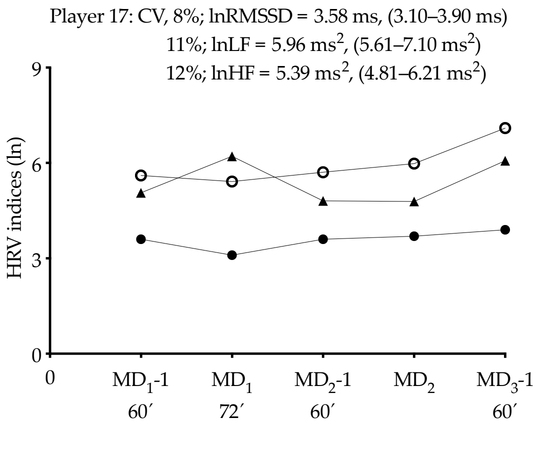
Player 17: CV, 8%; lnRMSSD = 3.58 ms, (3.10–3.90 ms)
11%; lnLF = 5.96 ms2, (5.61–7.10 ms2)
12%; lnHF = 5.39 ms2, (4.81–6.21 ms2)
HRV indices (ln)
9
6
3
0
0
MD1-1
MD1
MD2-1
MD2
MD3-1
60´
72´
60´
60´

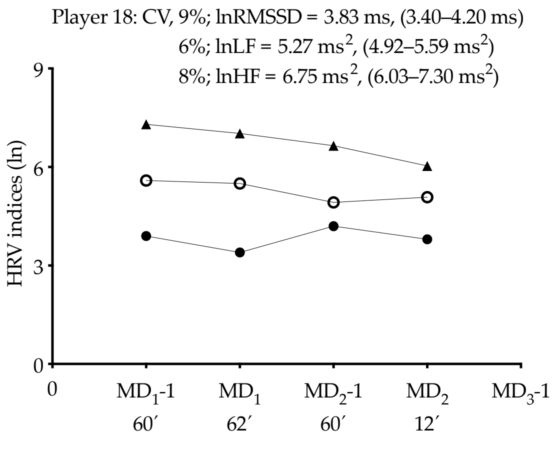
Player 18: CV, 9%; lnRMSSD = 3.83 ms, (3.40–4.20 ms)
6%; lnLF = 5.27 ms2, (4.92–5.59 ms2)
8%; lnHF = 6.75 ms2, (6.03–7.30 ms2)
HRV indices (ln)
9
6
3
0
0
MD1-1
MD1
MD2-1
MD2
MD3-1
60´
62´
60´
12´
