# Supplementary figures and images for: Sleep Indices and Cardiac Autonomic Activity Responses during an International Tournament in a Youth National Soccer Team
Source: Int J Environ Res Public Health. 2021 Feb 20;18(4):2076. doi: 10.3390/ijerph18042076 (PMC7924379; doi:10.3390/ijerph18042076)

## Supplement file 1.

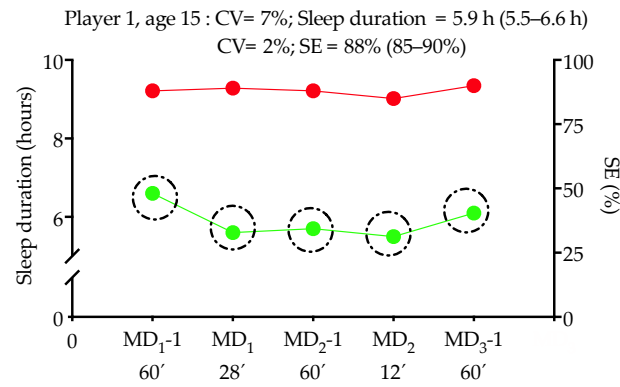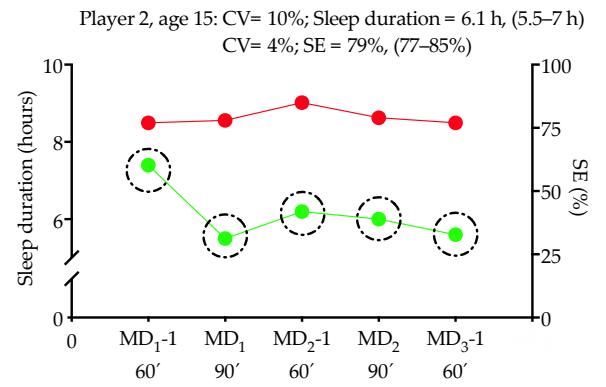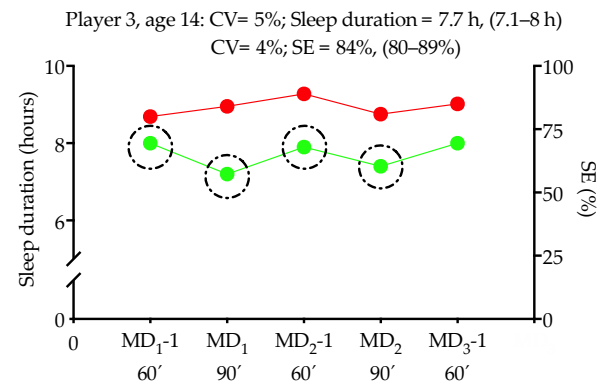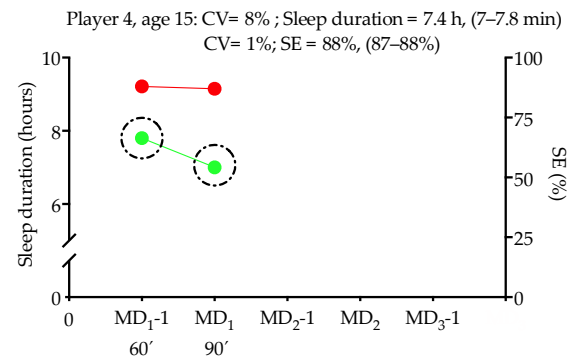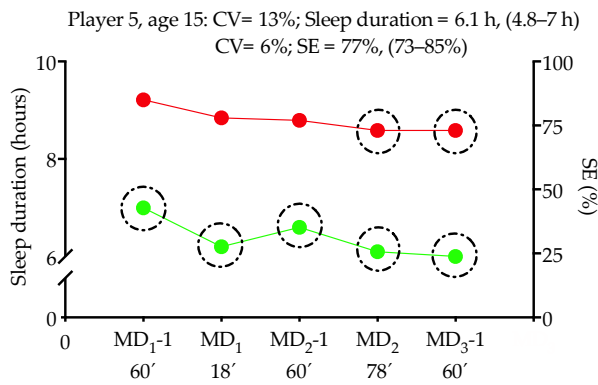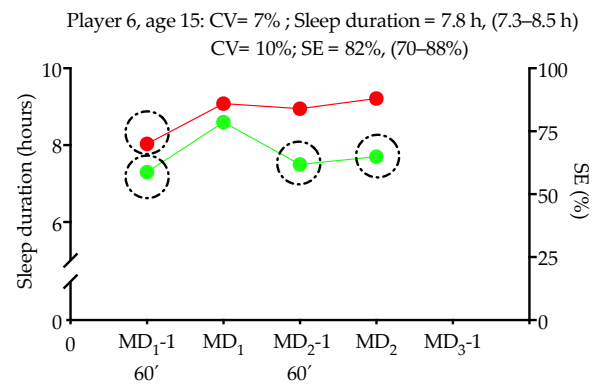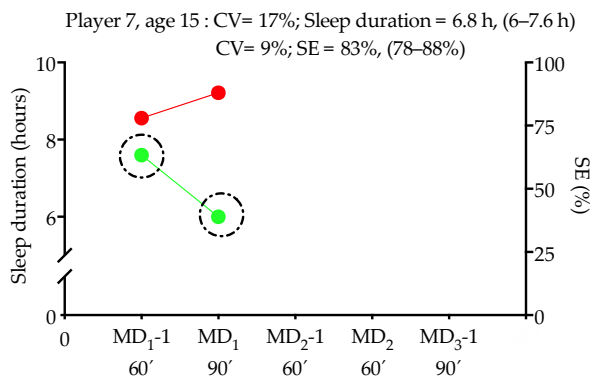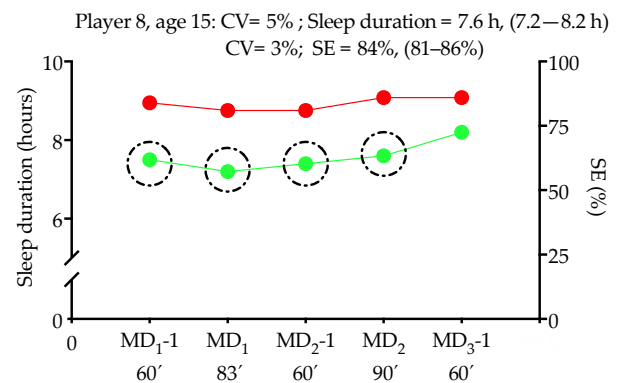

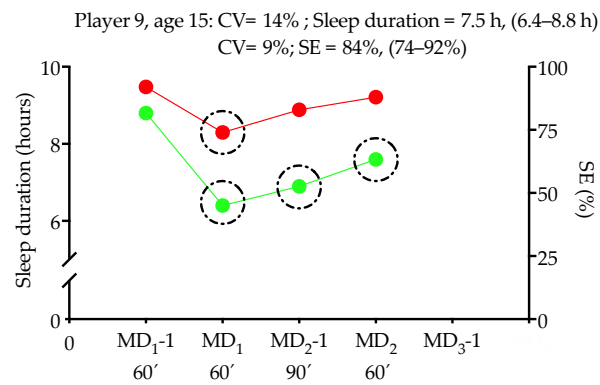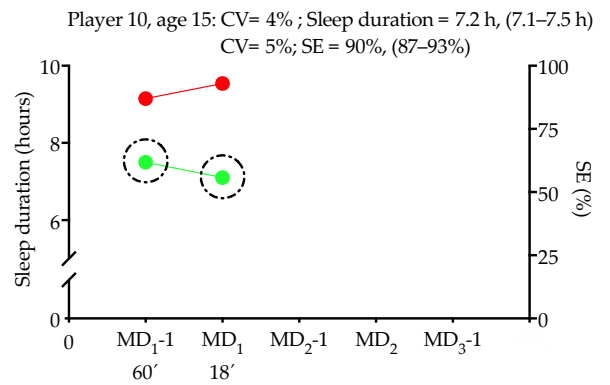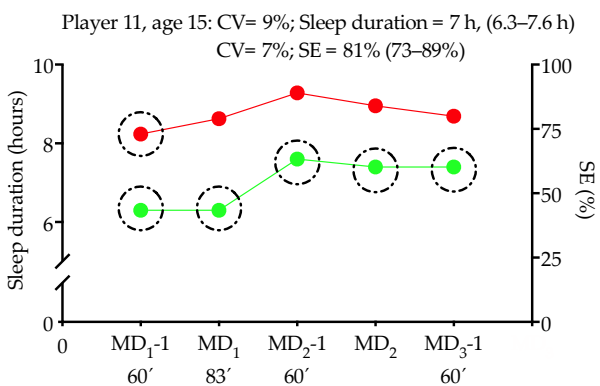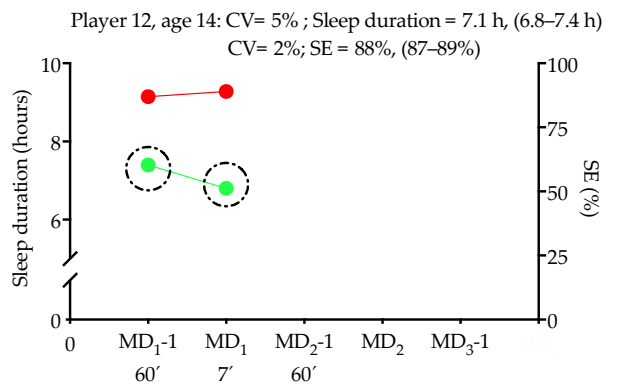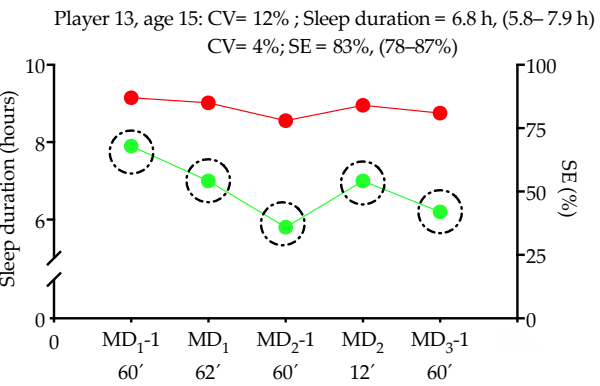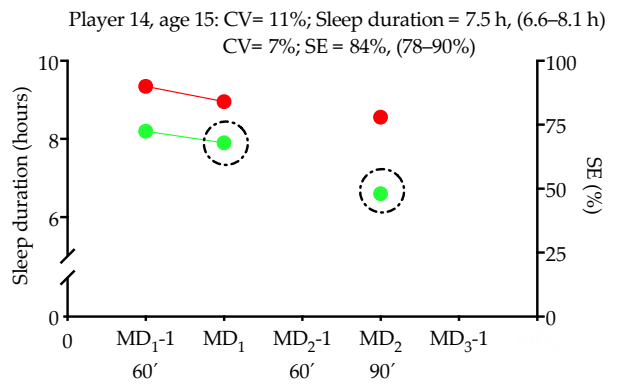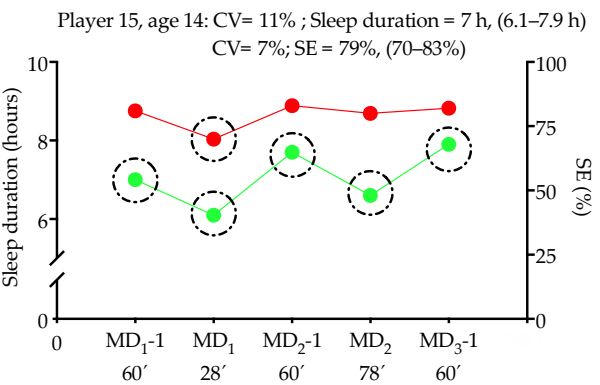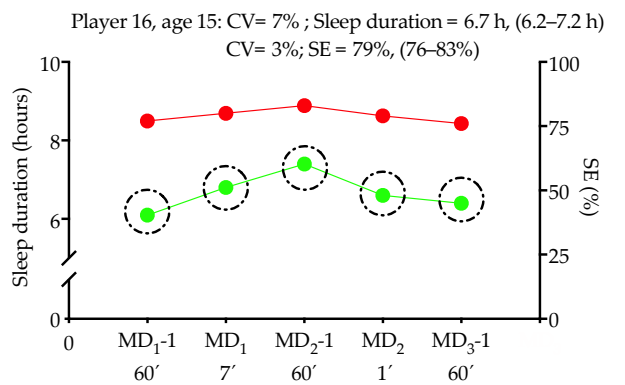

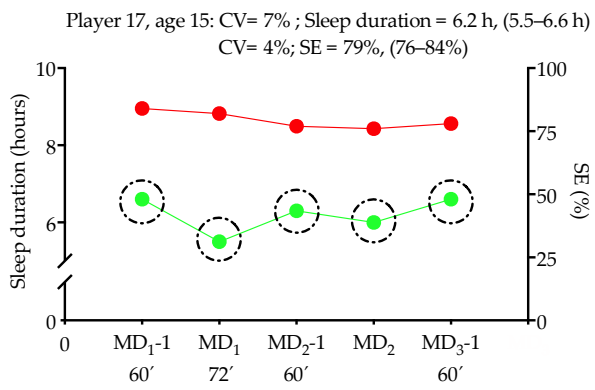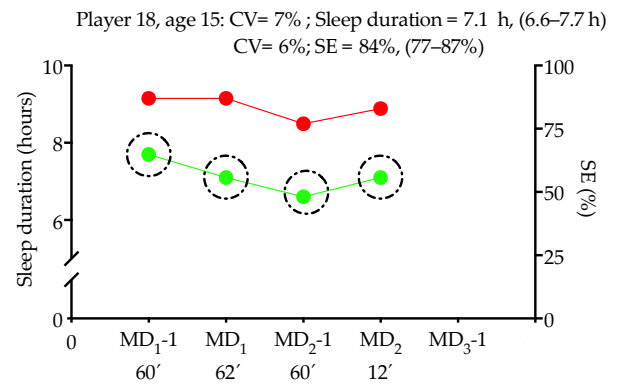

Supplement: Supplementary file 1 [file ijerph-18-02076-s001.zip › Supplement file 1.pdf]
